# Hearing loss and use of health services: a population-based cross-sectional study among Finnish older adults

**DOI:** 10.1186/s12877-016-0356-5

**Published:** 2016-11-08

**Authors:** Tuija M. Mikkola, Hannele Polku, Päivi Sainio, Päivikki Koponen, Seppo Koskinen, Anne Viljanen

**Affiliations:** 1 Gerontology Research Center and Department of Health Sciences, University of Jyvaskyla, P.O. Box 35, Viveca, Jyvaskyla, 40014 Finland; 2 Folkhälsan Research Center, Helsinki, Finland; 3 National Institute for Health and Welfare, Helsinki, Finland

**Keywords:** Hearing loss, Health services, Health services needs and demand, Pure-tone audiometry, Aged, Aging

## Abstract

**Background:**

Older adults with hearing difficulties face problems of communication which may lead to underuse of health services. This study investigated the association of hearing loss and self-reported hearing difficulty with the use of health services and unmet health care needs in older adults.

**Methods:**

Data on persons aged 65 and older (*n* = 2144) drawn from a population-based study, Health 2000, were analyzed. Hearing loss was determined with screening audiometry (*n* = 1680). Structured face-to-face interviews were used to assess self-reported hearing difficulty (*n* = 1962), use of health services (physician and nurse visits, health examinations, mental health services, physical therapy, health promotion groups, vision test, hearing test, mammography, PSA test) and perceived unmet health care needs. Multivariable logistic regression analyses were used.

**Results:**

After adjusting for socio-economic and health-related confounders, persons with hearing loss (hearing level of better ear 0.5–2 kHz > 40 dB) were more likely to have used mental health services than those with non-impaired hearing (OR = 3.2, 95 % CI 1.3–7.9). Self-reported hearing difficulty was also associated with higher odds for mental health service use (OR = 2.1 95 % CI 1.2–3.5). Hearing was not associated with use of the other health services studied, except presenting for a hearing test. Persons with self-reported hearing difficulty were more likely to perceive unmet health care needs than those without hearing difficulty (OR = 1.7, 95 % CI 1.4–2.1).

**Conclusions:**

Older adults with hearing loss or self-reported hearing difficulty are as likely to use most health services as those without hearing loss. However, self-reported hearing difficulty is associated with experiencing unmet health care needs. Adequate health services should be ensured for older adults with hearing difficulties.

**Electronic supplementary material:**

The online version of this article (doi:10.1186/s12877-016-0356-5) contains supplementary material, which is available to authorized users.

## Background

Hearing loss is relatively rare in young adults but its prevalence rises exponentially in older population [1] due to age-related sensorineural hearing loss [2]. It has been reported that in the United States more than 50 % of adults aged 70 and older and over 80 % of persons aged 80 and older have hearing loss [1, 3]. Due to the aging of the population, the number of persons with hearing loss is expected to rise substantially in the coming years.

The diagnosis and treatment of hearing loss may require several visits to different health care professionals, such as a general practitioner, otorhinolaryngologist and audiologist. On the other hand, for a marked proportion of those with hearing loss, the condition remains undiagnosed, and even after diagnosis does not receive rehabilitation [4]. Certain diseases, such as cardiovascular diseases [5] and diabetes [6], may increase the risk of developing hearing loss. The treatment of these diseases, although not a result of hearing loss per se, may lead to higher use of health services in this group. Further, hearing loss may also indirectly increase the need of other health services, as hearing loss elevates the risk for developing cognitive impairment [7], depression [8] and disability in activities of daily living [9]. The association between hearing loss and use of health services may also be confounded by sociodemographic factors. Higher age [10, 11] and low socioeconomic status [5, 11–13] are risk factors for both hearing loss and poor health, which in turn increase the need of health services. Male gender is another risk factor for hearing loss [11] and gender has been shown to have complex effects on health and health care use over the life-course [14, 15].

On the other hand, persons with hearing loss may avoid contacts with health care personnel owing to communication problems. It has been shown that older persons suffering from hearing problems are socially less active, i.e. participate in various social activities less often and meet other people less often than persons without hearing problems [16, 17], most probably due to the distress caused by difficulties in communication [18]. Similar distress and avoidance behavior may also apply to visits to health care practitioners, leading to lower health service use by persons with hearing loss.

Little is known about whether older adults with hearing loss use health services less or more than others. Only one hearing loss study has focused on older adults (who turned 65 during the year of the study) [19]. The authors found that hearing loss was associated with increased odds of making a visit to a health care practitioner. The result remained significant even after excluding hearing-related visits, suggesting that persons with hearing loss also use more health services other than those needed specifically for treating their hearing loss. Although the authors adjusted for chronic conditions, they did not adjust the analyses for socioeconomic factors which may confound the association between hearing and use of health services. Previous studies with a wide age range, from adolescence or young adulthood to old age, have found a higher than average utilization of physician services among persons with hearing loss; however, these studies did not control for socioeconomic status [20] or diseases that may confound the association between hearing and use of health services [21, 22]. One previous study on adults below age 65 adjusted for socioeconomic and health-related confounders and found significantly more contacts with health care among persons with hearing loss than among persons with normal hearing [23]. However, when hearing-related visits were excluded the difference disappeared. This suggests that, apart from those related directly to their hearing loss, persons with hearing loss do not use more health services than other people. In summary, it appears that the previous studies on the associations between hearing loss and use of health services have controlled for confounding factors to a varying extent and that the results are contradictory.

One explanation for the differences in the results of the previous studies may lie in the use of either objective or subjective methods for measuring hearing. It has been shown that objective and subjective measures of hearing do not correlate very highly and they cover partly different aspects of hearing performance [24]. Pure-tone audiometry, most widely used objective assessment of hearing, measures mostly peripheral sensory functioning in optimal conditions whereas subjective measures are influenced by personal factors and everyday acoustic environment of a person. Therefore, it is important to study both objective and subjective measures of hearing simultaneously.

Despite adjusting use of health services for the needs variables (i.e. diseases) persons may vary in perceived access to, and adequacy of, health services. Persons with hearing problems may be dissatisfied with the quality of health services due to communication problems with health care providers and hence, they may experience unmet health care needs. Consequently, in addition to absolute use of health services, perceived unmet need should be studied. To the best of our knowledge, only one study has investigated the association between hearing problems and perceived unmet health care needs. The results showed that, among Canadians from 12 years to old age, persons who reported hearing problems also reported more unmet health care needs than those not reporting hearing problems [20].

In previous studies investigating the associations between hearing and health services, the participants have mainly been either younger adults [23] or drawn from a wide age range [20–22], making it hard to draw conclusions concerning older adults. Further, previous studies have not explored objectively measured and self-reported hearing simultaneously. Therefore, more knowledge is needed on whether older adults with hearing loss use health services more or less than those without hearing loss and whether they perceive that health services meet their needs. The purpose of the present study was to explore the associations of hearing loss, determined by both audiometry and self-report, with the use of various health services and perceived unmet health care needs among Finnish adults aged 65 and older.

## Methods

The Health 2000 Study, conducted in 2000–2001, is a cross-sectional survey comprising a comprehensive health interview and a detailed health examination in a population-based large sample of Finnish adults [25]. The sampling was carried out using two-stage stratified cluster sampling. The sample consisted of 8028 adults aged 30 and older, and was representative of the same-age Finnish general population. In the oldest age group, 80 and older, the sampling probability was doubled to ensure a sufficient number of the adults in this group were included in the sample. The present analysis was confined to adults aged 65 and older (range 65 to 99 years, *N* = 2 144). The sample participation rate for those aged 65 and over was 89 % for the interviews, and 83 % for the health examination.

Computer-assisted personal health interviews (questions available at http://www.terveys2000.fi/forms.html) were carried out at the participants’ homes or in residential institutions. In the interviews, the interviewer read the questions from the laptop screen and entered the answers directly into the laptop. Health examinations were performed in public health centers or in temporary examination facilities. If the participant was unable to travel to the health center or temporary facility, an abbreviated examination was carried out in the participant’s home [25].

### Assessment of hearing

Pure-tone air-conduction hearing thresholds were assessed for both ears without a hearing aid using a screening audiometer (Micromate 304, Madsen Electronics) at the frequencies of 0.5, 1 and 2 kHz in a silent room [25]. Headphones with padded earpieces were used to minimize any environmental noise. The lowest signal intensity was 5 dB. The test started from the better-hearing ear or the right ear (if the participant reported no difference between the ears) at a frequency of 1 kHz (at 25 dB, or more for older people and those who seemed hard of hearing). Intensity was then reduced in decrements of 10 dB until the participant could no longer hear the signal. The intensity was then increased by in increments of 5 dB until the participant was able to hear the signal. The lowest intensity that the participant could hear was determined as the hearing threshold at 1 kHz. Next, hearing thresholds at frequencies of 2 and 0.5 kHz were similarly assessed, after which the other ear was assessed. If the participant could not hear at the intensity of 90 dB, 99 dB was marked as the hearing threshold. Better ear hearing level (BEHL_0.5–2kHz_) was calculated as the mean value over the measured frequencies. Test-retest repeatability was excellent (intraclass correlation coefficient = 0.97) [25]. Hearing level was dichotomized by categorizing those with BEHL_0.5–2kHz_ > 40 dB as having hearing loss and those with BEHL_0.5–2kHz_ ≤ 40 dB as having no hearing loss. A hearing threshold of 40 dB is the lower limit for moderate hearing loss [26] and was chosen as the cut-off since mild hearing loss (26 to 40 dB) is unlikely to markedly affect communication situations in health care, as these mostly take place between two persons in fairly quiet surroundings. Audiometric data were available for 1680 (78 %) persons.

Self-reported hearing difficulty was assessed with the question “Can you hear without difficulties what is said in a conversation between several people (with or without a hearing aid)?”. The response categories were 1) I can hear without difficulties 2) I can hear, but it causes difficulties and 3) I cannot hear at all. The latter two categories were combined as the number of men in the third category was too low to permit analysis of some of the outcome measures. Data on self-reported hearing difficulty was available for 1962 (92 %) persons.

### Use of health services

In Finland, public health services, funded by local and central government, are available to all citizens. Some services (e.g. prevention, such as cancer screening) are free of charge and for some services (e.g. physician’s services, physiotherapy), small fees are charged. If a citizen uses private health services a minor proportion of the costs is usually borne by the Social Insurance Institution of Finland. Audiologic rehabilitation, including fitting a hearing aid, is provided free of charge by the public specialized health care service after referral from primary health care.

As a part of the home interview, the participants were asked how many times during the last 12 months they had visited a physician (not including hospitalizations) at a health center (primary care), hospital outpatient clinic (secondary care), occupational health care clinic, private clinic, in connection with a home visit, or elsewhere. The numbers of visits reported were summarized. Participants who reported hearing loss were also asked to state the number of physician visits within the last 12 months due to their hearing loss. This number was subtracted from the total number of physician visits to yield the number of visits not related to hearing loss. The total number of visits to a nurse within the last 12 months was obtained from three distinct questions (occupational nurse; other nurse; home visits). As physician visits (median 2, minimum 0, maximum 50), physician visits due to hearing loss (median 2, minimum 0, maximum 50) and nurse visits (median 0, minimum 0, maximum 1095) were non-normally distributed they were categorized. Physician visits and physician visits due to hearing loss were classified into categories no visits/1–4 visits/5 or more visits) and nurse visits were classified into categories no visits/1–5 visits/6 or more visits. The proportion of the participants reporting a high number of nurse visits was larger than the proportion reporting a high number of physician visits. Applying cut points of five for physician visits and six for nurse visits yielded comparable distributions between these variables. Participants were also asked whether they had received physical therapy (via a referral from a physician) and whether they had used mental health services within the last 12 months. They were further asked whether they had undergone any health examinations (organized within occupational health care; for war veterans; related to the driver’s license; in connection with unemployment; or for other any reasons) within the last 5 years. Participants were also asked to report any vision test, hearing test, mammography, or prostate-specific antigen (PSA) test taken during the last 5 years. The mammography analysis included women younger than 70, as they form the target group for the mammography screening arranged by municipal public health services. Participation in health promotion groups was defined as having attended a group targeting weight management, smoking cessation, neck/back rehabilitation, other physical exercise, mental wellbeing, support for patients’ relatives, or problems with alcohol or other addictions, parenthood, self-care/management of an illness, or other health promoting activities within the last 5 years. The participants were further asked: “Do you have a chronic illness for which you would like to get continuous treatment by a doctor but do not receive it?” and “Do you have a chronic condition for which you would like to get other type of care but do not receive it?”. Participants answering yes to either question were considered to experience unmet health care needs.

### Potential confounders

Potential confounders were selected according to the criteria suggested by McNamee [27]. Accordingly, a confounder must be a cause of the outcome (use of health services), be correlated with the exposure (hearing loss/difficulty) and not be affected by the exposure. Sociodemographic factors included age, sex, mother tongue (Finnish/Swedish/other), income, years of education, and living alone (yes/no). Age and sex were obtained from the population register and income from taxation records. Household net income was divided by the number of consumption units in the household (first adult with weight 1, other adults 0.7 and children under 18 years 0.5) to yield the participant’s income. Self-reported diseases and health behavior that have been found to be associated with hearing loss, namely cardiovascular disease (myocardial infarction, angina pectoris, hypertension, lower limb arterial embolism) [5], stroke [28], arthritis (rheumatoid or osteoarthritis) [29], diabetes [30], alcohol use (8+ units/week vs. less) [31] and smoking (former or current vs. never) [32] were obtained from the home interview and self-administered questionnaire. Hearing aid use was defined as daily or almost daily use, and was based on two questions: “Do you have a hearing aid?” (yes/no) and “Do you use it daily or almost daily?” (yes/no). For calculation of body mass index (BMI), body weight and height were measured using standard procedures. If measured data were not available for a participant, self-reports were used to calculate BMI. Binocular far vision acuity was assessed on a decimal scale with eyeglasses on (if the participant usually wore them) using an illuminated (>350 lx) letter chart (Precision Vision Letter Chart Acuity Tests) [33]. Far vision acuity was dichotomized into the categories <0.5 (low vision), corresponding to <20/40 in 20/20 scale, and ≥0.5 (normal vision) [33].

### Data analysis

The sampling design, i.e. stratification and clustering, was taken into account in all analyses. Observations were weighted to reduce bias due to non-response and to correct for oversampling of those aged >80 years using inverse probability weights constructed using register data on geographical area (university hospital and health center district), age, sex and mother tongue [25]. As age has a strong effect on hearing loss, background characteristics for those with and without a hearing loss were calculated controlling for the effect of age. *P*-values for comparisons were obtained from age-adjusted logistic and linear regression analyses using Stata version 14. Continuous variables were standardized for the analyses. Hearing loss was found to have significant interactions with sex on physician visits, physical therapy, and hearing test. Self-reported hearing difficulty showed a significant interaction with sex on physical therapy. Therefore, the results on these outcomes are reported separately for men and women.

First, we analyzed the proportions of health service users, adjusted for sex (in case of no interaction) and age, using logistic regression analysis and the predictive margins function in Stata. In the case of ordinal regression analysis, the odds ratio describes how likely persons with hearing loss are to have a certain value (versus all the lower values) of the ordinal outcome variable compared to those without hearing loss. Then, multivariable-adjusted logistic regression models were run using MPlus version 7 [34]. In the multivariable-adjusted models, sociodemographic variables and hearing aid use were used as covariates for all the outcome variables. In addition, diseases, smoking, alcohol use, and BMI were used as additional covariates for physician and nurse visits and participation in a health promotion group. For use of physical therapy, cardiac diseases, stroke, and rheumatoid arthritis/osteoarthritis were the additional covariates. For vision examination, far vision, diabetes and stroke were the additional covariates. Breast cancer and prostate cancer were used as additional covariates for mammography and the PSA test, respectively. The same analyses were repeated with self-reported hearing difficulty as the main predictor. Hearing aid use was not entered into the model since persons who had a hearing aid were asked to evaluate their hearing when wearing the hearing aid. The analyses employed the maximum likelihood estimator which automatically takes into account missing data in the dependent variables but not in the independent variables. Auxiliary variables were not used in the maximum likelihood estimation.

Next, the maximum likelihood method was applied in another way to further test that the results were not biased by missing data in the independent variables. This was done by repeating the above mentioned regression analyses and simultaneously requesting means and variances for the independent variables and using Monte Carlo integration without analysis weights in MPlus. In this procedure, the analysis takes into account missing data also in the independent variables. Maximum likelihood method does not fill in missing values but uses observed data to estimate the parameters of the variables with missing data. Based on the available data of the variables in the regression model (complete and incomplete), it identifies parameter estimates that have the highest likelihood of underlying the observed data. The maximum likelihood method, along with multiple imputation, is among the two missing data analysis techniques that are considered as efficient for accounting for missing data when the data are missing at random or missing completely at random [35]. Even if the data is not missing at random maximum likelihood yields less biased estimates than deletion techniques [35].

## Results

### Characteristics

In total, 43 % (*n* = 837) of the participants (*n* = 1962) reported difficulty hearing a conversation between several people. Among those tested by audiometry (*n* = 1680), the weighted prevalence of hearing loss (BEHL_0.5–2kHz_ > 40 dB) was 16 % (non-weighted *N* = 328) (Table 1). Persons with hearing loss were older (mean 79 SE .13 vs. 72 SE .39 years, *p* < .001) than those without hearing loss. When adjusted for age, persons with a hearing loss (*n* = 328) had lower education (79 % vs. 72 % in the lowest tertile, *p* = .029), income (34 % vs. 26 % in the lowest tertile, *p* = .004) and BMI (mean 27 vs. 28 kg/m^2^, *p* = .047), and were more likely to have low vision (16 % vs. 12 %, *p* = .034) than persons without hearing loss (*n* = 1352). Of the persons with hearing loss, 33 % reported using a hearing aid daily while of those without hearing loss 0.8 % used a hearing aid daily (*p* < .001). Hearing aid users were present among those categorized as without hearing loss (<40 dB) as the category also included persons with mild hearing loss (25–40 dB).

**Table 1:** Background characteristics of the study participants for the whole sample and according to hearing status

	All, *N* = 2144		No hearing loss (BEHL_0.5–2kHz_ ≤ 40 dB), *N* = 1352		Hearing loss (BEHL_0.5–2kHz_ > 40 dB), *N* = 328		
	Mean^a^	SE^a^	Mean^a^	SE^a^	Mean^a^	SE^a^	*p* ^b^
BEHL_0.5–2kHz_ (dB), *n* = 1680	26	0.37	21	.25	52	1.0	<.001
Body mass index (kg/m^2^), *n* = 1867	27	0.12	28	.13	27	.30	.047
	*N*	%^a^	*N*	%^a^	*N*	%^a^	*p* ^c^
Male, *n* = 2144	766	39	515	39	108	45	.063
Language, *n* = 2144							.338
Finnish	1739	87	1237	92	283	90	
Swedish	150	6.7	96	7.1	40	8.7	
Other	299	5.8	19	1.3	5	1.6	
Lives alone, *n* = 1864	892	44	570	43	172	41	.648
Education, *n* = 1980							.029
Highest	180	9.3	136	10	24	7.0	
Middle	329	17	247	18	37	14	
Lowest	1471	73	966	72	264	79	
Income, *n* = 2144							.004
Highest tertile	729	36	533	39	65	30	
Middle tertile	730	35	470	35	109	36	
Lowest tertile	729	29	349	26	154	34	
Smoker, *n* = 1891	637	36	491	37	87	37	.999
Alcohol use ≥8 units/week, *n* = 1742	114	7.5	105	8.3	9	6.6	.489
Cardiovascular disease, *n* = 1985	1168	59	812	60	192	60	.990
Stroke, *n* = 1993	171	7.7	92	6.7	35	8.0	.427
Diabetes, *n* = 1996	256	12	159	12	52	15	.236
Arthritis, *n* = 1989	895	45	594	44	167	50	.067
Low vision, *n* = 1689	298	14	173	12	111	16	.034
Hearing aid user, *n* = 1975	118	5.1	12	0.8	94	33	<.001

*BEHL* better ear hearing level threshold

^a^Weighted and age-adjusted

^b^ *p*-value for comparison between Hearing loss and No hearing loss from linear regression analysis

^c^ *p*-value for comparison between Hearing loss and No hearing loss from logistic regression analysis

### Use of health services

Table 2 presents the proportions of health service users for persons with and without hearing loss adjusted for age, and also for sex in cases where there was no interaction between sex and hearing loss. Men with hearing loss were more likely to have visited a physician five times or more (31 % vs. 21 %) and less likely to not have visited a physician (17 % vs. 25 %) during the last 12 months (*p* = .020). Persons with hearing loss were less likely to have participated in a health promotion group during the last 5 years than persons without hearing loss (16 % vs. 23 %, *p* = .009). Both men (46 % vs. 29 %, *p* = .003) and women (42 % vs. 13 %, <.001) with hearing loss were more likely to have been for a hearing test during the last 5 years than those without hearing loss. In the multivariable-adjusted logistic regression analyses, hearing loss was associated with increased odds for having used mental health services within the last 12 months (OR 3.2, 95 % CI 1.3–7.9, *p* = .034). Women with hearing loss were more likely to have been for a hearing test within the last 5 years than women without hearing loss (OR 3.4, 95 % CI 2.3–5.2, *p* < .001). When maximum likelihood method was used to account for missing data in both independent and dependent variables, the results were parallel to the original results (mental health services OR 2.7, 95 % CI 0.9–7.2, *p* = .052; hearing test among women OR 2.5, 95 % CI 1.6–4.1, *p* < .001) (Additional file 1). Hearing loss was not associated with use of the other health services studied or unmet need for health care. We also ran supplementary analyses to see whether the results remained unchanged when a pure-tone hearing level of 25 dB was used as the cut point. Women with at least mild hearing loss (>25 dB) were more likely (OR 1.7, 95 % CI 1.2–2.4, *p* = .010) to have been for a hearing test than those with normal hearing (≤25 dB) whereas the associations between mild hearing loss and use of the other health services studied were statistically nonsignificant (Additional file 2).

**Table 2:** Proportions of health service users and odds ratios for hearing loss explaining use of health services

	Age-sex adjusted proportion %				Multivariable adjusted odds ratio		
	All	no HL, *N* = 1352	HL, *N* = 328	p for no HL vs. HL	OR	95 % CI	p
Physician visits, all (last 12 months), *n* = 1925							
Men	25/52/22^a^	25/54/21^a^	17/52/31^a^	.020	1.3^c^	0.8;2.2	.328
Women	19/57/24^a^	18/58/24^a^	17/58/26^a^	.695	1.0^c^	0.7;1.3	.882
Physician visits, not related to hearing loss (last 12 months), *n* = 1914	22/55/22^a^	21/57/22^a^	19/56/24^a^	.257	1.0^c^	0.7;1.3	.981
Nurse visits (last 12 months), *n* = 1980	55/26/19^b^	55/27/18^b^	51/29/20^b^	.291	1.2^c^	0.9;1.7	.261
Health examination (last 5 years), *n* = 2006	41	44	40	.382	0.7	0.5;1.1	.201
Mental health service (last 12 months), *n* = 1907	2.2	2.0	4.4	.059	3.2	1.3;7.9	.034
Physical therapy (last 12 months), *n* = 1909							
Men	10	9.9	15	.172	2.0^d^	1.0;3.9	.088
Women	14	15	12	.298	0.9^d^	0.5;1.5	.744
Health promotion group (last 5 years), *n* = 1677	22	23	16	.009	0.8^c^	0.6;1.2	.385
Vision test (last 5 years), *n* = 1882	64	67	62	.153	0.7^e^	0.5;1.0	.085
Hearing test (last 5 years), *n* = 1870							
Men	32	29	46	.003	1.1	0.6;2.0	.759
Women	17	13	42	<.001	3.4	2.3;5.2	<.001
Mammography (women <70 year, last 5 years), *n* = 259	63	64	65	.973	1.7^f^	0.3;10.5	.624
PSA test (men, last 5 years), *n* = 628	28	29	25	.485	1.0^g^	0.5;1.8	.896
Unmet health care needs, *n* = 1929	26	26	32	.100	1.3	0.9;1.7	.232

Results are given separately for men and women where interaction of sex is significant

Only models that include both sexes are adjusted for sex. All multivariable adjusted models are controlled for age, mother tongue, living alone, income, education, and hearing aid use

*HL* hearing loss, better ear hearing threshold level 0.5–2 kHz > 40 dB; *OR* odds ratio

^a^Proportions for 0/1–4/5+ visits

^b^Proportions for 0/1–5/6+ visits

^c^Model additionally adjusted for diseases, smoking, alcohol use, and BMI

^d^Model additionally adjusted for cardiovascular diseases, stroke, and arthritis

^e^Model additionally adjusted for far vision, diabetes and stroke

^f^Model additionally adjusted for breast cancer

^g^Model additionally adjusted for prostate cancer

Table 3 presents the proportions of health service users for persons with and without self-reported hearing difficulty adjusted for age, and also for sex in cases with no interaction between sex and self-reported hearing difficulty. Persons with self-reported hearing difficulty were more likely to have visited a physician five times or more (26 % vs. 22 %) and less likely to not have visited a physician (20 % vs. 24 %) during the last 12 months than persons reporting no hearing difficulty (*p* = .020). Further, persons with hearing difficulty were more likely to have been for a hearing test (29 % vs. 19 %, *p* < .001) and more likely to perceive unmet need for health care (32 % vs. 21 %, *p* < .001) compared to those reporting no hearing difficulty. In the multivariable-adjusted logistic regression analyses, self-reported hearing difficulty was associated with increased odds for having used mental health services during the last 12 months (OR 2.1, 95 % CI 1.2–3.5, *p* = .025) and the odds for having been for a hearing test within the last 5 years (OR 1.8, 95 % CI 1.4–2.3, *p* < .001). Hearing difficulty also increased the likelihood of reporting perceived unmet needs for health care (OR 1.7, 95 % CI 1.4–2.1, *p* < .001). When maximum likelihood method was used to account for missing data in both independent and dependent variables, the results were parallel to the original results (mental health services OR 2.0, 95 % CI 1.1–3.6, *p* = .029; hearing test OR 2.0, 95 % CI 1.5–2.5, *p* < .001; unmet health care needs OR 1.7, 95 % CI 1.3–2.1, *p* < .001) (Additional file 3). Self-reported hearing difficulty was not associated with physician visits not related to hearing loss, nurse visits, health examinations, physical therapy, health promotion group, vision test, mammography or PSA test.

**Table 3:** Proportions of health service users and odds ratios for self-reported hearing difficulty explaining use of health services

	Age-sex adjusted proportions %			Multivariable adjusted odds ratio		
	no HD, *N* = 1125	HD, *N* = 837	*p*	OR	95 % CI	*p*
Physician visits, all (last 12 months)	24/55/22^a^	20/55/26 ^a^	.020	1.2^c^	1.0;1.5	.072
Physician visits, not related to hearing loss (last 12 months)	24/55/21 ^a^	21/55/24 ^a^	.112	1.1^c^	0.9;1.4	.291
Nurse visits (last 12 months)	56/26/18 ^b^	53/27/20 ^b^	.109	1.1 ^c^	0.9;1.4	.280
Health examination (last 5 years)	40	40	.736	1.1	0.9;1.3	.655
Mental health service (last 12 months)	1.7	3.1	.058	2.1	1.2;3.5	.025
Physical therapy (last 12 months)						
Men	8.8	12	.145	1.3^d^	0.8;2.1	.298
Women	14	14	.826	1.1^d^	0.8;1.5	.647
Health promotion group (last 5 years)	22	22	.728	0.9^c^	0.7;1.1	.518
Vision test (last 5 years)	65	65	.979	1.0^e^	0.8;1.2	.709
Hearing test (last 5 years)	19	29	<.001	1.8	1.4;2.3	<.001
Mammography (women <70 year, last 5 years)	64	64	.946	1.1^f^	0.6;2.0	.870
PSA test (men, last 5 years)	28	28	.950	0.9^g^	0.7;1.2	.608
Unmet health care needs	21	32	<.001	1.7	1.4;2.1	<.001

Results are given separately for men and women where interaction of sex is significant. Only models that include both sexes are adjusted for sex. All multivariable adjusted models are controlled for age, mother tongue, living alone, income and education

*HD* self-reported hearing difficulty, *OR* odds ratio

^a^Proportions for 0/1–4/5+ visits

^b^Proportions for 0/1–5/6+ visits

^c^Model additionally adjusted for diseases, smoking, alcohol use, and BMI

^d^Model additionally adjusted for cardiovascular diseases, stroke, and arthritis

^e^Model additionally adjusted for far vision, diabetes and stroke

^f^Model additionally adjusted for breast cancer

^g^Model additionally adjusted for prostate cancer

As hearing loss was not associated with most of the studied health services we explored whether this was due to hearing aid use accounting for a major part of the covariance between hearing loss and use of health services. This was not the case, as hearing aid use was significantly associated only with the likelihood of having been for a hearing test in both men (OR 6.6, 95 % CI 3.5–12.6, *p* < .001) and women (OR 4.7, 95 % CI 2.6–8.4) and not associated with the other health services studied or perceived unmet needs for health care.

## Discussion

The results of the present analysis showed that older adults with hearing loss or self-reported hearing difficulty were as likely to use most health services as other older adults. However, both groups were more likely to have used mental health services and to have been for a hearing test. Nevertheless, persons with self-reported hearing difficulty were more likely to experience unmet needs for health care than persons reporting no hearing difficulty.

The higher use of mental health services within the last 12 months in persons with a hearing loss or self-reported hearing difficulty is in line with previous research showing more mental health service use among war veterans with hearing loss aged 18 and older [36]. A meta-analysis of longitudinal studies showed that persons with a hearing loss have increased risk for depression [37], although this has not been confirmed by subsequent longitudinal studies [38, 39]. However, in the present study, adding mental illness as a covariate did not reduce the strength of the association, implying that the effect was not mediated through manifest mental illness (data not shown). Instead, persons with hearing loss may have had subclinical depressive symptoms for which at least some had received mental health services. It should be noted, however, that a very low proportion (2.3 %) of the present participants had used mental health services within the last 12 months. This is considerably less than the prevalence of any depressive disorder (6.7 %) in Finnish adults older than 65 [40].

Unexpectedly, after adjusting for socioeconomic factors and hearing aid use, hearing loss was associated with having been for a hearing test only among women. However, in both sexes hearing aid use was associated with increased likelihood of having been for a hearing test. Men were more likely than women to have been for a hearing test among both those with hearing loss and those without hearing loss. The pattern was similar with regard to self-reported hearing difficulty. Others have also reported that men are more likely to have their hearing tested than women [4]. These results suggest that men are more frequently referred for a hearing test than women irrespective of actual hearing loss or self-reported hearing difficulty. The authors speculate that the sex difference may partly derive from job history. Men are more likely to be exposed to occupational noise [41], which may lead to more follow-up of hearing in older men.

The present study showed that persons with self-reported hearing difficulty were more likely to perceive unmet health care needs compared to those reporting no hearing difficulty, although they were equally or more likely to have used health services. In the present study, owing to the general nature of the question posed, unmet needs may not be related solely to hearing health services but also include other health services. Our finding in older adults is in line with those of a previous study among Canadians across a very wide age range in which the likelihood of unmet health care needs among those with self-reported hearing difficulty was 1.3 times higher than among those who did not report hearing problems [20].

Interestingly, only self-reported hearing difficulty, but not measured hearing loss, was associated with unmet health care needs in the present study. We speculate that the explanation may lie in differences in communication strategies, which are intrinsically taken into account in the self-reported hearing question but not in the measured pure-tone hearing levels. Effective communication strategies may alleviate the negative influence of hearing loss on speech understanding [42] and thereby reduce self-reported hearing difficulty. Further, the question on self-reported hearing treated all those with hearing problems equally, irrespective of hearing aid use. This means that if a person perceived communication difficulties, with or without a hearing aid, he was classified as having hearing difficulty whereas the analysis of measured hearing, adjusted for hearing aid, treated all hearing aid users as a uniform group. Perceived unmet needs for health care may be explained by greater dissatisfaction with the availability of specialist health care and follow-up treatment of older adults with hearing loss than those without disabilities [43] although evidence has also been shown for similar levels of satisfaction with access between persons with and without hearing loss [44]. Unmet needs of and dissatisfaction with health care may result from a patient with hearing problems receiving inadequate information owing to communication problems with the health care provider [45].

This study has several strengths. First, the sample was population-based with a relatively high participation rate, which increases the generalizability of the results. Second, a wide variety of health services and perceived access to health services were analyzed. Third, the use of both objective and subjective hearing assessments provides a deeper understanding on whether use of health services is more dependent on physiological hearing loss or perceived hearing. On the one hand, objective hearing assessment may reveal hearing problems even if the person has not noticed hearing loss himself. On the other hand, of two persons with equal physiological hearing loss one may find hearing problems more disabling due to environmental and personal factors. Fourth, we adjusted the analyses for several important factors, i.e. certain comorbidities and socioeconomic status, that are likely to confound the association between hearing and use of health services.

The present study also has some limitations. Those who declined participation in the health examination, including audiometry, in the present study were less likely to have used some of the health services studied (visits to a physician, health examination, physical therapy, vision test, mammography) than those who participated. They also had poorer hearing according to a subjective evaluation by the interviewer, although their self-reported hearing was similar to that of those who participated in the health examination (data not shown). Due to this selection bias, the results may underestimate the strength of the association between measured hearing and use of health services. This study did not include telephone contacts with health care providers or health services provided via the internet. Omitting telephone contacts from the analysis may have led the use of health services to be more underestimated in persons without hearing loss than in those with hearing loss. This is because those with hearing loss are more likely to avoid telephone contacts. However, omission of internet health services is not likely to have presented a significant bias as such services were very uncommon in Finland at the time of the data collection. We measured hearing only at frequencies 0.5, 1 and, 2 kHz. As age-related hearing loss typically first affects high frequencies (4 kHz and higher), it is likely that we classified some persons with hearing loss (determined on the basis of frequencies 0.5–4 kHz) as without hearing loss. However, the definition of hearing loss in this study is likely to have identified persons who experience problems when communicating with health care providers. Although we used several sociodemographic and health-related variables to control for potential confounding factors, we cannot totally rule out the possibility that residual confounding partially explains some of the findings. Further, self-reported health service use as the outcome variable may introduce recall bias. Finally, the cross-sectional design of the present analysis limits the drawing of causal inferences.

## Conclusions

Self-reported hearing difficulty or moderate pure-tone hearing loss do not appear to be associated with the use of most health services among older adults. However, higher use of mental health services in persons with self-reported hearing difficulty or moderate pure-tone hearing loss, independently of socioeconomic status and confounding comorbidities, suggests that mental health screening may be justified for older adults with hearing problems. Further, self-reported hearing difficulty in men and women and moderate hearing loss in women were associated with higher likelihood of having had a hearing test. Despite similar or higher use of health services, persons reporting hearing difficulty perceived more unmet needs for health care than those without hearing difficulties. One possible explanation for unmet needs for health care may be communication difficulties between patients and health service providers, which could be alleviated by aural rehabilitation and/or by educating health care providers on effective communication strategies. Nevertheless, experience of unmet needs for health care in persons with hearing difficulties may indicate insufficient care of their health problems. This in turn may lead to further health impairment of persons with hearing difficulties. Hence, it seems justified to pay special attention to ensuring that an older patient reporting hearing difficulty receives adequate health services. Further studies are needed to explore whether hearing problems predict future health service use and to explore the factors underlying unmet health care needs in older adults with hearing problems. This study covered only a part of health care among older persons with hearing problems. Future studies concerning use of medication and community services could expand understanding on how older persons with hearing problems take care of their health and manage with their everyday life.

## Additional files

Additional file 1: Table on the associations between hearing loss and health service use in older adults when accounting for missing data with maximum likelihood. Multivariable adjusted odds ratios (OR) for hearing loss explaining use of health services when accounting for missing data with maximum likelihood method (*N* = 2144). (PDF 211 kb)
Additional file 2: Table on health service use in older adults with (pure-tone hearing level > 25 dB) and without hearing loss. Age-sex adjusted proportions person with (*N* = 787) and without hearing loss (better-ear hearing level at frequencies 0.5–2 kHz >25 dB, *N* = 893) and multivariable-adjusted odds ratios for hearing loss explaining use of health services. (PDF 234 kb)
Additional file 3: Table on the associations between self-reported hearing difficulty and health service use in older adults when accounting for missing data with maximum likelihood. Multivariable adjusted odds ratios (OR) for self-reported hearing difficulty explaining use of health services when accounting for missing data with maximum likelihood method (*N* = 2144). (PDF 6 kb)

## Abbreviations

BEHL: Better ear hearing level
CI: Confidence interval
dB: decibel
HD: Hearing difficulty
HL: Hearing loss
kHz: kilohertz
OR: Odds ratio
PSA: Prostate-specific antigen
SE: Standard error
