# Supplementary material for: Hearing loss and use of health services: a population-based cross-sectional study among Finnish older adults
Source: BMC Geriatr. 2016 Nov 8;16:182. doi: 10.1186/s12877-016-0356-5 (PMC5100231; doi:10.1186/s12877-016-0356-5)
Supplement: Additional file 1: — Table on the associations between hearing loss and health service use in older adults when accounting for missing data with maximum likelihood. Multivariable adjusted odds ratios (OR) for hearing loss explaining use of health services when accounting for missing data with maximum likelihood method (N = 2144). (PDF 211 kb) [file 12877_2016_356_MOESM1_ESM.pdf]

Additional file 1

Multivariable adjusted odds ratios (OR) for hearing loss explaining use of health services when accounting for missing data with maximum likelihood method (N=2144).

| | **OR** | **95%CI** | **p** |
|---|---|---|---|
| Physician visits, all (last 12 months) | | | |
| Men | 1.3[c] | 0.7;2.3 | .351 |
| Women | 0.9[c] | 0.7;1.3 | .734 |
| Physician visits, not related to hearing loss (last 12 months) | 1.0[c] | 0.8;1.4 | .899 |
| Nurse visits (last 12 months) | 1.0[c] | 0.7;1.5 | .825 |
| Health examination (last 5 years) | 0.8 | 0.5;1.2 | .220 |
| Mental health service (last 12 months) | 2.7 | 1.0;7.2 | .052 |
| Physical therapy (last 12 months) | | | |
| Men | 1.8[d] | 0.8;4.0 | .130 |
| Women | 0.8[d] | 0.5;1.4 | .523 |
| Health promotion group (last 5 years) | 0.8[c] | 0.6;1.2 | .366 |
| Vision test (last 5 years) | 0.8[e] | 0.5;1.1 | .124 |
| Hearing test (last 5 years) | | | |
| Men | 1.5 | 0.8;2.8 | .240 |
| Women | 2.5 | 1.6;4.1 | <.001 |
| Mammography (women <70 yrs, last 5 years) | 1.5[f] | 0.2;10.6 | .683 |
| PSA test (men, last 5 years) | 1.0[g] | 0.5;2.1 | .997 |
| Unmet health care needs | 1.1 | 0.8;1.6 | .432 |

Hearing loss, better ear hearing threshold level 0.5-2kHz>40dB.

Results are given separately for men and women where interaction of sex is significant.

Only models that include both sexes are adjusted for sex. All multivariable adjusted models are

controlled for age, mother tongue, living alone, income, education, and hearing aid use.

[c]Model additionally adjusted for diseases, smoking, alcohol use, and BMI.

[d]Model additionally adjusted for cardiovascular diseases, stroke, and arthritis.

[e]Model additionally adjusted for far vision, diabetes and stroke.

[f]Model additionally adjusted for breast cancer.

[g]Model additionally adjusted for prostate cancer.
