# Supplementary material for: Hearing loss and use of health services: a population-based cross-sectional study among Finnish older adults
Source: BMC Geriatr. 2016 Nov 8;16:182. doi: 10.1186/s12877-016-0356-5 (PMC5100231; doi:10.1186/s12877-016-0356-5)
Supplement: Additional file 2: — Table on health service use in older adults with (pure-tone hearing level > 25 dB) and without hearing loss. Age-sex adjusted proportions person with (N = 787) and without hearing loss (better-ear hearing level at frequencies 0.5–2 kHz >25 dB, N = 893) and multivariable-adjusted odds ratios for hearing loss explaining use of health services. (PDF 234 kb) [file 12877_2016_356_MOESM2_ESM.pdf]

Additional file 2

Proportions of person with (better-ear hearing level at frequencies 0.5-2 kHz >25 dB, N=787) and without hearing loss (N=893) and multivariable-adjusted odds ratios for hearing loss explaining use of health services. Results are given separately for men and women where interaction of sex is significant.

	**Age-sex adjusted proportion %**				**Multivariable adjusted odds ratio**		
	**no HL_{25dB}**	**HL_{25dB}**	**p**		**OR**	**95%CI**	**p**
Physician visits, all (last 12 months)							
Men	26/53/21[a]	22/54/25[a]	.287		0.9[c]	0.6;1.3	.705
Women	18/58/24[a]	16/58/26[a]	.357		1.1[c]	0.8;1.5	.561
Physician visits, not related to hearing loss (last 12 months)	21/57/22[a]	20/57/23[a]	.585		0.9[c]	0.7;1.3	.616
Nurse visits (last 12 months)	58/26/16[b]	49/29/21[b]	.005		1.3[c]	1.0;1.6	.112
Health examination (last 5 years)	44	42	.411		0.9	0.7;1.1	.321
Mental health service (last 12 months)	2.1	2.7	.496		1.3	0.7;2.4	.477
Physical therapy (last 12 months)							
Men	8.2	14	.034		1.8[d]	1.1;3.0	.056
Women	15	14	.721		1.0[d]	0.7;1.3	.853
Health promotion group (last 5 years)	24	20	.054		0.9[c]	0.7;1.2	.515
Vision test (last 5 years)	66	66	.791		1.0[e]	0.8;1.3	.958
Hearing test (last 5 years)							
Men	27	40	.004		1.4	0.9;2.1	.159
Women	13	23	<.001		1.7	1.2;2.4	.010
Mammography (women <70 yrs,	64	65	.911		1.4[f]	0.6;3.4	.524

last 5 years)							
PSA test (men, last 5 years)	31	25	.238		0.8[g]	0.5;1.3	.388
Unmet need for health care	26	29	.222		1.1	0.9;1.3	.635

Notes. HL_{25dB}=hearing loss, better ear hearing threshold level 0.5-2kHz>25dB.

Only models that include both sexes are adjusted for sex. All multivariable adjusted models are controlled for age, mother tongue, living alone, income, education, and hearing aid use.

[a]Prevalences for 0/1-4/5+ visits

[b]Prevalences for 0/1-5/6+ visits

[c]Model additionally adjusted for diseases, smoking, alcohol use, and BMI.

[d]Model additionally adjusted for cardiovascular diseases, stroke, and arthritis.

[e]Model additionally adjusted for far vision, diabetes and stroke.

[f]Model additionally adjusted for breast cancer.

[g]Model additionally adjusted for prostate cancer.
