# Supplementary material for: Hearing loss and use of health services: a population-based cross-sectional study among Finnish older adults
Source: BMC Geriatr. 2016 Nov 8;16:182. doi: 10.1186/s12877-016-0356-5 (PMC5100231; doi:10.1186/s12877-016-0356-5)
Supplement: Additional file 3: — Table on the associations between self-reported hearing difficulty and health service use in older adults when accounting for missing data with maximum likelihood. Multivariable adjusted odds ratios (OR) for self-reported hearing difficulty explaining use of health services when accounting for missing data with maximum likelihood method (N = 2144). (PDF 6 kb) [file 12877_2016_356_MOESM3_ESM.pdf]

Additional file 3

Multivariable adjusted odds ratios (OR) for self-reported hearing difficulty explaining use of health services when accounting for missing data with maximum likelihood method (N=2144).

	OR	95%CI	p
Physician visits, all (last 12 months)	1.2[c]	1;1.5	0.078
Physician visits, not related to hearing loss (last 12 months)	1.1[c]	0.9;1.4	0.312
Nurse visits (last 12 months)	1.0[c]	0.8;1.3	0.750
Health examination (last 5 years)	1.1	0.9;1.4	0.499
Mental health service (last 12 months)	2.0	1.1;3.6	0.029
Physical therapy (last 12 months)			
Men	1.4[d]	0.8;2.3	0.234
Women	1.1[d]	0.7;1.5	0.764
Health promotion group (last 5 years)	1.0[c]	0.7;1.3	0.767
Vision test (last 5 years)	1.0[e]	0.8;1.2	0.843
Hearing test (last 5 years)	2.0	1.5;2.5	<.001
Mammography (women <70 yrs, last 5 years)	1.1[f]	0.5;2.1	0.847
PSA test (men, last 5 years)	0.9[g]	0.7;1.2	0.539
Unmet health care needs	1.7	1.3;2.1	<.001

Results are given separately for men and women where interaction of sex is significant. Only models that include both sexes are adjusted for sex. All multivariable adjusted models are controlled for age, mother tongue, living alone, income and education.

[c]Model additionally adjusted for diseases, smoking, alcohol use, and BMI.

[d]Model additionally adjusted for cardiovascular diseases, stroke, and arthritis.

[e]Model additionally adjusted for far vision, diabetes and stroke.

[f]Model additionally adjusted for breast cancer.

[g]Model additionally adjusted for prostate cancer.
